# Perceptions of Spanish-language COVID-19 video messaging among the Hispanic community: A qualitative study in the United States of America

**DOI:** 10.1371/journal.pone.0339634

**Published:** 2026-07-29

**Authors:** Grace Ellen Brannon, Karishma Chatterjee, Chyng-yang Jang, Charla Markham Shaw, Vinicio Sinta, Julian Rodriguez

**Affiliations:** Department of Communication, University of Texas at Arlington, Arlington, Texas, United States of America; Public Library of Science, UNITED KINGDOM OF GREAT BRITAIN AND NORTHERN IRELAND

## Abstract

With evidence linking credible and quality COVID-19 vaccine information to positive vaccination attitudes and intentions, it's vital to understand how communities facing health disparities access health information about COVID-19. This interview study of 49 Hispanic individuals in a large southern suburban county is a part of a larger three-year multi-method study. Through the lens of self-determination theory, this project investigates perceptions of four television-aired Spanish-language video messages on CseOVID-19 vaccination developed from findings of prior local focus groups and panel surveys supported by community partners. The present study’s findings show human-centered, gain-frame messaging was positively received and highlight the need for targeted Spanish-language public health messaging in Spanish news media to improve quality information about COVID-19. Gentler suggestions for health protective behaviors may foster greater perceived resonance, credibility, and informational value of public health messages, with potential implications for psychological reactance and subsequent responses.

## Background

The official COVID-19 national and public health emergencies were terminated in May 2023. From October 1, 2024 through September 27, 2025, the Centers for Disease Prevention and Control (CDC) estimated 14–20.7 million COVID-19 illnesses, 3.4–4.8 million outpatient visits, 390,000–550,000 hospitalizations, and 45,000–64,000 deaths in the United States of America (USA) [1,2]. This surge placed a heavy financial burden for both individuals and healthcare systems [3]. Yet, despite this considerable disease burden, a substantial number of adults remain hesitant about COVID-19 vaccination, limiting the potential of COVID-19 vaccines for preventing severe outcomes as well as reducing associated financial strains. Vaccine hesitancy has been linked to concerns around the pace of initial authorization, side effects, and long-term effects exacerbated by conspiracy theories stemming from misinformation, contributing to lower-than-expected vaccination rates particularly among those facing disproportionate physical, psychological, and socioeconomic impacts in part due to reduced access to healthcare [4,5]. U.S. Hispanics continue to have low rates of insurance coverage, especially in states with limited subsidies for health access such as Texas where the percentage of uninsured Hispanics stood at 28.1 percent in 2022 [6]. As indicated in previous publications [7], since the late 20th century, broad terms like *Hispanic* or *Latina/o/x* have been used in the United States to refer to people of Latin American heritage. Traditionally, masculine terms such as *Latino* and *Chicano* have served as gender-neutral labels following Spanish grammar conventions; however, some scholars use *Latina* and *Chicana* specifically to refer to women. Additionally, terms like *Latinx* and *Latine* are employed to intentionally include identities outside the male/female gender binary [8]. For clarity and consistency, this article uses the pan-ethnic term *Hispanic*, as it was the word most frequently mentioned by participants when describing their community and ethnic identity [9].

In the current post-emergency era, reduced public involvement in boosting COVID-19 preventative measures puts the onus of acquiring regular vaccinations on individuals, caregivers, and other community stakeholders. In the face of growing evidence of vaccine information as a factor associated with pro-vaccination attitudes and intentions to vaccinate [10,11], it is crucial to assess how communities with a higher rate of exclusion from healthcare access acquire and disseminate vaccine-related information. Improving public health media initiatives for the Hispanic population (e.g., through Spanish-language messaging) is one recommended method of closing information gaps [12]. Therefore, we relied on the self-determination theory to provide the theoretical lens for this project.

### Self-determination theory

Self-determination theory (SDT) describes how individuals who experience self-determination (i.e., through having choice-making abilities and control over one’s life) feel motivated to act and improve their health outcomes [13–15]. According to SDT, if one is self-determined, then one is motivated to take specific action. Four specific needs are at play: autonomy, relatedness, competence, and beneficence. First, autonomy describes people’s need to be free to make their own decisions; in the context of COVID-19 vaccines, this may manifest in people not wanting to be told what to do and wanting credible information to make their own choices. Second, relatedness highlights the need to be respected and understood [16]. People tend to agree with sources they feel connected to and trust [17] underlining the importance of culturally appropriate messaging. Third, competence describes the importance of quality information; as without feeling competent and having quality information to act on, people may not know what to do or feel ready to receive a COVID-19 vaccine. Fourth, beneficence demonstrates the newest established need of making positive impacts on others, which can be both at the individual and community levels; these are particularly important for Hispanic families when considering cultural traits like *familismo*, the heavy consideration given to family welfare in health decision-making [18].

### Framing vaccination decision-making

The way health information is framed affects audiences’ cognitions, attitudes, and behaviors (e.g., social distancing) [19,20]. These include the *loss* and *gain* frames, as well as the *individual* versus *collective* frames. The loss/gain binary refers to the highlighting of positive vs. negative outcomes of a posited health-related action [21], while the individual and collective frames look at prospective actions as a private decision or as one that could be impactful for the broader community [22].

SDT may help guide health-related decisions. When loss frames are used in a way that supports autonomy (e.g., by allowing individuals to make their own choices about how to avoid the negative outcomes associated with the losses), enhances competence (e.g., by providing clear strategies to prevent negative outcomes), and fosters relatedness (e.g., by highlighting social consequences and/or support), the loss frames can potentially drive more internalized, self-determined motivation. Conversely, gain-framed messages, which focus on the positive outcomes of a specific action, may be more effective when they align with an individual’s intrinsic goals and support the same psychological needs. Thus, the effectiveness of loss or gain framing is amplified when the message design is attuned to the SDT components [13,16,17], fostering motivation. To better understand this dynamic, it is important to examine these psychological needs and how they shape marginalized individuals’ perceptions of health messaging within specific cultural and linguistic contexts. Therefore, we ask:

*RQ1: What roles do autonomy, relatedness, competence, and beneficence play in participants’ evaluation of video-based messaging aired on Spanish-language news media about COVID-19 vaccination?*

### Information availability, quality, and spanish language considerations affecting vaccination intention or hesitation

Health literacy and access to up-to-date, scientifically based health information are part of social determinants of health [23,24], with health information gaps, mis/disinformation, and connectivity divides being among the main concerns during the COVID-19 emergency [25]. A study examining factors associated with COVID-19 vaccine uptake among Latino/a individuals living near the USA/Mexico border showed individuals were more likely to have received a COVID-19 vaccine if they had higher levels of trust in credible sources of health information (e.g., healthcare provider, US government) than those who did not trust these sources [26].

Uncertainty and a perceived lack of quality information are the most common reasons stated by those reluctant or opposed to getting a vaccine [27]. Similarly, concerns about the availability, clarity, and accuracy of vaccine information were the most common concerns related to COVID-19 vaccinations [28,29]. The nature of information needs, however, can be nuanced by a priori attitudes toward vaccines in a “continuum of hesitancy” [30]. While some participants framed a lack of information as hindrance to an informed decision, others with more negative vaccine attitudes focused on perceived censorship and other conspiratorial beliefs. Further, those who reported greater trust in faith leaders or social media contacts were less likely to have had a COVID-19 vaccine compared with those who did not trust those sources [26].

COVID-19-related information needs are high among the Hispanic population, but longstanding gaps in the availability and accessibility of health information persist, particularly for Spanish-language speakers [31]. Misinformation is prolific in conversations about COVID-19 and is particularly prevalent in online Spanish-language conversations [31], social networks, and news sources [32]. Misinformation can increase one’s hesitancy to receive a vaccine, particularly if the myths are not combated in an accurate, culturally appropriate way

Spanish-language messaging is, therefore, of great importance. However, despite federal guidance recommending information be disseminated in multiple languages, COVID-19 information was not equivalently provided in Spanish [33]. Additionally, few news outlets report Spanish-language news. Participants in one qualitative study found Spanish-language television news covers COVID-19 vaccine information too quickly and does not provide in-depth coverage [7]. Combined, these issues limit the accuracy and quantity of information accessible to Spanish-speaking households. Given research recommends Spanish-language media as a method to diminish the COVID-19 vaccination gap, we ask,

*RQ2: How did Hispanic community participants perceive video-based messaging aired on Spanish-language news media addressing their information needs about COVID-19 vaccination?*

Therefore, the goal of this manuscript is to investigate how ethnically minoritized individuals in a large county in Texas perceived video-based messaging aired on Spanish-language news media about COVID-19 vaccination. Specifically, the goal is to understand how individuals within these communities perceived acceptability, credibility, and engagement, in efforts to inform the development and implementation of targeted interventions that address vaccine hesitancy within these communities.

## Materials and methods

### Project overview

This project is the culmination of a three-year multi-stage project employing several methodologies including community engagement, focus groups, four panel survey rounds, news report development, and individual interviews. Focus group findings are published [7]. We used the consolidated criteria for reporting qualitative studies (COREQ-32) checklist [34].

### Participants

Interviewees were recruited from participants of bilingual panel surveys about COVID-19 messaging and health information needs conducted in 2023. To be eligible for the panel surveys (and the interviews that led to this specific corpus of data), participants were 18 + years, self-identified as Hispanic and lived or worked in the county of interest. A total of 49 (37 women and 12 men) participated in semi-structured interviews conducted in the first quarter of 2024.

### Procedures

The interview activities described in this study were reviewed by the Institutional Review Board (IRB) at the University of Texas at Arlington (Protocol #2022−0072). The IRB determined that these activities do not meet the definition of human subjects research under 45 CFR 46.102(l) and do not require IRB review and approval. Specifically, the IRB confirmed that the project meets the definition of program evaluation and/or public health surveillance under 45 CFR 46.102(l)(2), consistent with CDC Policy 557, “Distinguishing Public Health Research and Public Health Nonresearch” as the activities were conducted as part of a CDC-funded community project locally designed to assess and improve public health [35]. Community members engaged in this project included local librarians and owners/operators/leaders of local Hispanic-owned businesses. Nine trained bilingual interviewers (six females, three males) conducted 49 interviews in private meeting rooms at three public county libraries. Participants provided implied informed consent by reading an information sheet and indicating verbal agreement before completing the interview. Interviews were offered on weekdays, weekends, morning, and evening times to maximize participation. While they were planned to last 45–50 minutes, some went on longer. Participants were compensated with a $100 retail chain gift card.

### Interview protocol

The project team developed a semi-structured interview guide to probe participants perceived information consumption and sharing habits, as well as their perception of Hispanic-oriented messaging (see S1 Text for news video report development; see S2 Text for the interview guide) regarding the COVID-19 vaccine. Participants were first shown the four videos aired as part of local Spanish-language television newscasts during 2022 and 2023. See Table 1 for topic, framing, and information about each video. After each, they were led to talk about their responses to the content and formatting of the reports. Interviewees were specifically asked if they knew the information in the news stories beforehand. Afterwards, participants were also prompted to discuss their experiences with COVID-19 vaccine information seeking, consumption, and sharing.

**Table 1:** Videos produced for the COVID-19 messaging project.

Video	Topic	Gain/Loss Frame Present	Individualistic/ Collectivistic Frame Present	Description
**1**	Multisystem Inflammatory Syndrome in children	Loss	Individual	A Hispanic male doctor was interviewed for this news report. The story was humanized by profiling a Hispanic family whose child suffered from MIS-C and recovered after months of treatment. The report was presented and narrated by a female reporter.
	Exemplar Quote			Doctor sound bite: “Symptoms of multisystem inflammatory syndrome include: Abdominal pain, diarrhea, vomiting, dizziness rashes, and high fevers lasting more than three to four days.”
**2**	“Long COVID”	Loss	Individual	A Hispanic female doctor was interviewed for this news report. The central compelling character (CCC) of the story was a Hispanic female suffering from long COVID. The story was presented and narrated by a female reporter.
	Exemplar Quote			Mariela Urtado, suffering from long COVID, sound bite: “I feel disoriented… I go outside and I get lost… that doesn’t happen all the time, only when I have strong headaches. It terrifies me... I don’t go out.”
**3**	COVID vaccine: benefits and side-effects	Gain	Collectivistic	A Hispanic female doctor was interviewed for this news report discussing how vaccination reduced hospitalization and, therefore, the risk of overloading our healthcare system. Some of the side effects of the COVID vaccines was also addressed..
	Exemplar Quote			Female doctor sound bite: “We as Hispanics, we have to defend ourselves. We like to be with family and therefore we should be wise. If we’re going to be around a lot of people, we should get vaccinated.”
**4**	COVID vaccines and seniors	Gain	Collectivistic	A Hispanic female public health expert was interviewed for this new report. The CCC was a senior Hispanic female who sees the vaccine as a way to safely enjoy time with her family. The story was presented and narrated by a female reporter.
	Exemplar Quote			Female Hispanic senior sound bite: “We need to prevent diseases. We have to take care of ourselves so that our children… our grandchildren… can grow up healthy.”

All 49 interviews were audio recorded, transcribed, and when applicable, translated from Spanish to English using Adobe Premiere. The transcripts were manually reviewed for thoroughness and accuracy by members of the team. For Spanish-language interviews, questions related to transcription or translation were verified by Spanish-speaking members of the team.

We used two methods of validation in this study: prolonged engagement and triangulation (Creswell, 2007, p. 207−209). Five of the authors were involved in consistent field observation, including being present during the interviews, supporting student interviewers and building trust and rapport with participants, and providing clarification to interviewees as needed. During analysis, we used investigator triangulation: members of the research team independently coded transcripts, compared and discussed their interpretations of categories and themes, and resolved discrepancies through consensus. We also engaged in theoretical triangulation by iteratively comparing emerging themes with existing literature on COVID-19 communication and Hispanic health to refine our interpretations.

### Data analysis

Four study authors analyzed the interview data inductively and deductively through the constant comparative method [36], using SDT as a sensitizing framework allowing for identification and coding of recurrent patterns. This led to the establishment of categories and themes. After an initial round of open coding on a subset of transcripts, the team met to develop a preliminary codebook that included both data-driven codes and a small number of SDT-informed codes (e.g., autonomy, competence, relatedness). For example, we identified the concepts of autonomy and reactance as a pair of categories. Within this pair, the theme of choice was prevalent. Similarly, the category of storytelling matched that of relatedness. Within this category pair, the theme of welfare for the family was apparent. The third category pair was collective responsibility and beneficence. This pair reflected participants’ desire to look out for the larger family and community. Finally, in the category of competence, we found themes related to accurate and credible information. We then applied this codebook to the full dataset, iteratively refining code definitions and merging or splitting codes as needed across multiple coding cycles. At least two team members coded each transcript; disagreements were discussed in regular analytic meetings and resolved through consensus, with revisions to the codebook documented to preserve an audit trail. This process supports the dependability and confirmability of our findings.

## Results

Most participants stated overall positive, if cautious, attitudes toward COVID-19 vaccines and claimed to be active seekers of information about the virus and associated illnesses. They also described social media (i.e., Facebook, Instagram) as major sources of updates, as well as the main conduit to share information with their loved ones while acknowledging digital platforms as a source of misinformation. Many were aware of the role of ethnic media (i.e., Spanish-language television) as a key source of health information for older relatives and recent migrants. Our analysis centers SDT components of autonomy and reactance, relatedness and beneficence, and finally competence. See extended exemplars in Table 2.

**Table 2:** Participant exemplar quotes by theme.

Participant	Example Quote
**Theme: “You really have to make your own choice”: Autonomy and Reactance**	
JS	…I think they did a good job with this one, at least in terms of the questions that I have, because I think sometimes, too, you know, they're doctors and they're studying stuff. But you still don't have all the answers. So, you really have to make your own choice in terms of which direction you want to go with the vaccine. But you should still have access to information if you can.
IS	I feel like it's very important, but I'm also not going to push it on you, because everyone has to do something for themselves. There should be choice, but I feel like it's a very important thing to help save lives.
BA	I had actually never heard about MIS-C. I remember someone mentioning it, but I had never actually known the side effects. And my kids are five and eight… So for me, I was like “Hm, that's interesting.” I remember a family member talking about it, but I never looked into it. It's one of those things that… Because it's not your child, you kind of know it, but you don't, like, research it until your kids have symptoms of something and you're like “Let me go and find out about this sort of stuff so that my kids don't get sick.”
SC	…If I post it on my social networks I will be judged ‘crazy.’ Many times, no one looks at you, only that you are in the problem. Mhm. And when you're already in trouble, really, the least you want to do is publish.
LV	And I put it on my Facebook or send it to them on WhatsApp. Well, for my sisters, for my mom, so they can see it. But one time my sister told me, “You know what? Don't send those messages to my mom anymore, because you are stressing her out more.” When you talk to her, tell her “You know what? Take care of yourself, mom. Wash your hands…”
**Theme: “It just puts you in her shoes”: Relatedness and Beneficence**	
MM	The video shows how Hispanic people were affected at disproportionate rates, but most of us are also essential workers during that time. We were at the grocery store working or whatever business was open. Just for money. We were there as an employee putting ourselves on the line.
RR	…My grandma is kind of like that lady. She always wants us to be, you know, healthy. She's in Mexico, right now. But I'm sure if she was here, she would make sure that we were up to date with our vaccines. Especially now that I'm pregnant and, you know, I'm about to have my baby. I'm sure she would be reminding me “Make sure you're vaccinated” and stuff.
BA	…I feel like I can relate a little more because I got it. I was only tested three times, either at home or at the doctor, and on the fourth time I'm pretty sure I had it, because my kids had it and they were sleeping with me. We were taking care of them. […] I was fine, but after I got really sick, I lost a lot of hair and I still haven't been able to get it back. […] I would get tired when doing normal things, like if I wanted to vacuum or sweep the floors and mop in one go, I couldn't do it. I would just try to catch my breath and I couldn't do it.
RR	…I know how bad it is to have a sick kid. So I'm like, I learn a lot of stuff and I was able to kind of put myself in her situation. Being a mom…
JS	You know if I'm really thinking about it is just one is just how I don't feel like we as a community really do a lot of prevention. You know, we don't really go to the doctor very often unless we have to because we're sick… So really check-ups are not, like, at the top of our list, and I think vaccines fall within that, especially like COVID […] Now you probably have to get your insurance to pay for it.
ZP	I was the first one of my family to get COVID. I got it at work, and then everyone else got sick. I was like, I feel bad that I had given it to everyone. And they were all getting the symptoms. So when the vaccine came out, I got it instantly. […] I wanted to get to protect my family and everyone around me.
BL	Well, as far as having my own child... I’m scared, and just, you know… ways that I could possibly protect her. Which she had [or] does not have or had the shot. So, that's just something to kind of think about um still has again she's 11 so that is something that did have me um, frightened.
IS	…I feel like the stories about the family members, because it would make them think about their family… And my family is really big on family… So I feel like that would encourage them, and [also] the one with the babies, because I have a lot of little cousins and stuff. I feel like it would make them want to do it for their kids or their family...
LV	I sent this report to some of my friends on Facebook so that they could also see it, because they do not want to be vaccinated either. They say that this vaccine is harmful to us, that this vaccine is not true, that they just want to check our blood, that they want to control us, that who knows what? And now that they have become sick, I tell them “No, no, no. [That’s not right.]”
PW	For my parents, since they haven't gotten vaccinated… What would probably encourage them is my little girl. I don't want my little girl to get sick. And if my parents get sick, I don't want my girl to get sick. So that would be their [reason to get vaccinated].
LL	You can't blame them [family members]. You also have to take care of them. God, he makes doctors, doesn't he? Or does make those who make the vaccine, right? There are doctors and there are all kinds, because God gives them that you know, and God gives them that so that they learn and give it to us so that they can help us. I don't understand her [the niece] because she doesn't believe in it… it's that we all have to take care of each other, no matter what.
IS	I feel like she [grandmother in the news report] loved her family because for you to look for vaccinations near your home and not just get it for yourself. But you thought of everyone else and you thought about your grandkids and the people you like to like be around and cook for like. I felt like that was very selfless of her because some people don't think about that like they just do and they don't think about others.
**Theme: “I Feel Like I’m Late to the News”: Competence in Analyzing Information**	
IS	...I feel like I probably need to touch on more long-term information because I haven't really read a bunch, but I feel like I should still be educating myself. Even though I do believe in the vaccine. Like educate myself so I can educate others who don't know.
RT	…I didn't know anything. I just knew that COVID hit you. And like a flu, it hit you and went away and that was it… And that it would probably come back to you eventually. But I didn't know for sure that there were after-effects, that there were consequences. I didn't know that.
NC	…It's hard to know where to go to get the vaccine, and when you go like to Walmart or CVS or something it just says you know, COVID vaccine, but it doesn't say like “free”.
TW	It made me feel good knowing that, you know, they're trying to help with offering it for free. Um, because that would sway a lot of people right there.
MM	I wish I would have known about it more at the time. Because at the time all you saw were the deaths happening within older populations. I feel like that was overshadowing this. I'm very shocked to see that this was going on. I just thought if children had it, it was fine. They’re small kids, like, it was a cough or a cold to them. […] I feel like I'm late to this news.

### “You really have to make your own choice”: Autonomy and reactance

The first research question focused on components of SDT, specifically autonomy, relatedness, competence, and beneficence, and how they affected participants’ evaluations of the news report videos. Even when a clear majority of interview participants expressed enthusiasm for COVID-19 vaccines and needing more high-quality information about vaccination, their interpretation of their own role as health decision makers reflected a strong notion of individual autonomy. While healthcare professionals and scientists were often acknowledged as main sources of advice, both in the media and in person, some respondents highlighted their own role in putting health information to work on their own terms.

Many individuals prioritized autonomy, describing a hesitancy to intervene in their family or friends’ health decision-making practices, even if it posed a risk to others (IS, Table 2). Participants who were also parents reflected on the multiple layers of autonomy and the COVID-19 vaccine. These conversations were primarily focused around the first news report, where multisystem inflammatory syndrome in children (MIS-C) was discussed, and an image of a child on a ventilator was shown. Some participants found that image scary or distressing. However, participants discussed how little they knew about MIS-C, and the possibility that it could be their child(ren) to get sick highlighted the importance of information. One woman stated, “I didn't know that children were being affected like that. I don't think I had heard about MIS-C before. So, yes, this was new for me” [MM]. Participants wanted details about the long-term effects of MIS-C because it was part of their own research and decision-making process.

Other participants discussed considerations for sharing information with anti-vaccine family and friends. Feeling that one would be judged as “crazy” was a common fear, and others received pushback from family members (SC, LV, Table 2).

Many participants described family or friends’ reactance as influenced by the media they consumed. For example, MM said,

Vaccines are made for a reason. Yeah, there’s like not a conspiracy theory behind that I feel like it's very very important for everybody to be vaccinated. I think we saw it more in conservative media. How they were against masks, how they were against, um, vaccines. And I think they followed that type of media. They listen to that type of media.

### “It just puts you in her shoes”: Relatedness and beneficence

News videos containing relatable stories elicited relevant personal experiences and reflections from interviewees and clearly communicated the underlying relatedness motivation for vaccination. One video shown to participants generated the most expressions of relatability or identification with a source. Participants connected the story of a Hispanic grandmother planning to get the COVID-19 vaccine to safely meet her family at home to their own experiences with parents, grandparents, and other elderly relatives. This segment of the interviews led to some of the clearest expressions of affective engagement with the video reports and the underlying messaging about the importance of COVID-19 vaccines, with one stating “…[the] lady reminded me of my grandma. […] I was thinking about my grandma, mostly like, “Oh, yeah. She would do everything for us too.’” [ZP]. Others agreed, “Well, it reminded me of an older family member…Grandma or an aunt, somebody older, you usually go to their house. You eat, or they feed you. You hang around with them. […] I think it was more emotional.” [JS]

The identification with the main character in the news video was generally expressed in two ways. First, participants identified elderly relatives’ vulnerability as a factor in choosing to vaccinate, making comments like,

I can't really speak for everybody's family, but we're a really big family and we get together a lot… I have a lot of older people in my family like my oba and my tíos and we have like a lot of family members who get together. They're older, and sometimes they have health compromises. [IS]

Second, participants also identified family elders—and in this case, women—as key leaders and trendsetters in health decision-making.

It kind of hit home with that one, because speaking of your mother or grandmother, or someone that's an older family member and how to keep them safe or have they been vaccinated… Just to, you know, keep their health up to date, not for themselves, but for their children or grandchildren… [BL]

Further, the grandmother in the video reflected similar cultural values of familismo and how the participants had grown up. Participant NH stated, “They [production team] did a really good job with it… You know, abuelita is like the foundation of the whole family. So with her, you hear the grandma talking like your grandma.” This sentiment was echoed by other participants such as RR (Table 2).

While the video of the grandmother was almost universally relatable to participants, responses to other messages were more situational, though similarly powerful. MM described that “it's hard for us to stay away from family, which is a good thing. You know that we're like family oriented. But in this time [peak pandemic] that wasn't the time to… have family gatherings” and that the vaccine enabled family gatherings. The videos featuring a woman experiencing “long COVID” and a couple whose child had been diagnosed with MIS-C led participants to look back to their own experience with the pandemic or to speculate about their own role as caregivers (BA, RR; see Table 2).

Finally, some interviewees considered COVID-19 vaccine risks and implications perceived as specific to Hispanic communities. Several respondents addressed cultural traits like familismo and the importance of frequent family gatherings, and how there were amplified negative consequences of individuals ignoring preventative health measures at these family gatherings. Thinking about the virus and vaccine from the perspective of Hispanic identity also highlighted specific struggles worsened by persistent health inequities. For example, MM was sense-making about the presence of disproportionate COVID-19 rates because the Hispanic individuals occupied front line worker roles, and JS remarked on how the Hispanic community does not engage in health prevention practices (see Table 2). This awareness of community hardships was salient in other studies of Hispanic communities’ preferences regarding COVID-19 vaccination messaging (see Castellon-Lopez et al., 2022).

According to SDT, beneficence demonstrates the need to make positive impacts on others at individual and community levels, in other terms, collective responsibility. For Hispanic families, where family welfare is of the utmost importance in health decision-making, this was particularly prevalent (Katiria Perez & Cruess, 2014). Some participants recognized the need to look out for their family members broadly, saying, “We need to look out for ourselves. We need look out for our peers” [NH], while others focused on family members they felt responsibility for, such as children: “I told my children, ‘You know what? I don't receive people in my house who are not vaccinated’” [LV].

Many participants used their experience with COVID-19 and vaccines as a jumping off point to discuss their (or others’) collective responsibility in limiting the spread of the virus. JS shared receiving the vaccine out of concern for their mother and ZP felt guilty for passing on COVID-19 to family members, so when the vaccine became available, she got it (see Table 2). Of course, there were some participants whose family members (particularly parents) had yet to be vaccinated, and they considered persuasively framing the need to receive a COVID-19 vaccination in terms of looking out for other, more at-risk family members (PW, see Table 2)

Some participants reflected on instances where they tried to share information with their friends or family members to no avail. While they did engage in beneficence, ultimately, they recognized that there was nothing to be done to force the issue. LL described their niece as a churchgoing individual who refused vaccines. Navigating the tension of differences in family opinion**,** combined with religiosity, was challenging for them (see Table 2).

### “I feel like I’m late to the news”: Competence in analyzing information

The second research question focused on how participants perceived the news report videos as addressing their information needs about COVID-19 vaccine. These responses centered on needing accurate and credible information, particularly in a time of high uncertainty. CA described how videos should highlight physician interviews, “I think doctors should cover it more.” Many participants noted intention to do their own research about the COVID-19 vaccines. This desire to be self-informed is articulated by AA,

I think that you can never believe everything a hundred percent […] I am simply going to take my time to get informed, to enter the website, to see the reviews, to see what people are saying, to get informed. And once I gather information and I say “OK”, if it is credible, I am also going to check the vaccines.

In general, participants stated an interest in COVID-19 virus and vaccine information as a means for empowerment—a way to regain or maintain control in the face of continued uncertainty [37,38]. However, participants’ perceptions of information gaps are nuanced by personal experiences with COVID-19 as well as preexisting attitudes towards preventative measures like vaccination. The more enthusiastic among them, for instance, sought to stay updated to make informed decisions and support those who might need additional orientation or reassurance.

The timing of the interviews led many participants to discuss in retrospective health information practices and habits, which some indicated affected their ability to make decisions. The videos presented as part of the interview covered issues that maintained COVID-19 concerns at the forefront long after the initial contagion waves. Upon watching the videos, some participants reflected on how information gaps led to their previous assumptions and decisions related to the virus and vaccine. This was particularly the case for those whose life circumstances made them or their family especially vulnerable to COVID-19 related-illness (MM, Table 2).

Yet another group of participants framed the need for information in terms of personal empowerment; in some cases, this took the form of a retrospective analysis of their own responsibilities as parents, caregivers, or even just as the most informed among their family. In this context, the added certainty of updates about COVID-19 and its associated vaccine/s could act to minimize future risk and maintain control over their wellbeing and that of their loved ones. For participants with positive vaccine attitudes, practical, actionable advice on how, when, and where to secure boosters were especially valuable. CA said. “I have to get that [the vaccine], but they're not that accessible to my kids or to the adults. So, it's good to know that there are places that do give them for free.” It was highlighted as a strength in the video that provided information about the Bridge Access Program funding free vaccines for the uninsured but was identified as a shortcoming for the videos that talked about the benefits of vaccines without providing or highlighting the logistics, including the cost (NC, Table 2),

Another participant [TW] indicated that while they were anti-vaccine, even switching healthcare providers to avoid being “pushed” the recommended vaccines, it was still important for people to know where to find low-cost or free vaccines (See Table 2). Several participants who claimed to be vaccinated acknowledged having “come around” to getting the vaccine after initial skepticism in the face of scant information or what they deemed to be a *deficient information* campaign. For example, AJ said, “The one, probably really the only thing that I would feel comfortable with at the time was just more time, more studies on it and whatnot. But I ended up getting it.” Some discussed third-party vaccine hesitancy—that of family and friends—in terms of the questions that in their perspectives remained unanswered. Participants offered ways to improve the videos, by reporting details about boosters including their ingredients, like WR, “Maybe a little more information about… Like, she [the doctor] said that it's new every year, but you know, how they come up with the new formulas? […] How did they figure that it has to be different every year?”

## Discussion

Our interviews collected participants’ responses to Spanish language news stories focused on COVID-19 and related vaccines as well as reflections on their vaccination-related decisions and experiences. The analysis based on SDT offers valuable insights on health message considerations [15,16]. Relatedness was an illuminating factor among our participants. This insight is particularly relevant when addressing vaccine messaging to Hispanic communities; considering the importance of family as a motivator, as well as perspectives from individual circumstances (e.g., caregivers, parents, elderly), is key in encouraging specific health behaviors (e.g., COVID-19 vaccination) [39]. Our findings suggest a nuanced understanding and interpretation given the roles of generational perspectives, immigration status, and gender should be considered [40,41]. Indeed, while family consideration was strong, some participants prioritized autonomy such that they were hesitant to influence family and friend’s vaccine decision making.

These findings have implications for SDT as a theory of behavior change. Our participants’ experiences are reflected in one meta-analysis which found autonomous self-regulation (driven by intrinsic motivation and aligned with personal goals) promotes mental and physical health [16]. On the other hand, use of controlled regulation (driven by guilt, shame, or external factors) leads to short-term effect, anxiety, and dissatisfaction (p. 336). Therefore, practitioners promoting vaccine messaging must attempt to incorporate the concept of beneficence sans guilt or shame, particularly in efforts to avoid psychological reactance. Studies show that cultural contexts characterized by collectivist values like *familismo* commonly encourage group-based shame and guilt as responses to perceived transgressions against family expectations, functioning to restore group harmony but potentially increasing psychological distress when internalized excessively [42]. Further*, marianismo* emphasizes the roles Hispanic women should play in their family, especially related to responsibility and caregiving (particularly child and elder care). Hispanic women uphold this principle by prioritizing family health, supporting relatives, sharing health information, and facilitating group health behaviors—which can influence the entire household’s wellbeing [43,44]. Similarly, many participants in our study echoed the role of the grandmother in the family’s health decision making.

Responses to the videos indicated that different types of frames (e.g., both gain and loss frames) could be effective if properly contextualized [45]. Again, specific medical complications, underlying health conditions, and family considerations in the videos elicited strong reactions and made the frames more salient. Empirical evidence shows variation in success of using gain or loss frames messaging in the Hispanic community dependent upon the context (e.g., HPV vaccinations, healthy eating, physical activity) [46,47]. Our findings add to this research in that public health messaging aimed towards Hispanic communities may benefit from flexible framing strategies that are carefully adapted to the population’s lived experiences and cultural priorities. Specifically, both gain and loss frame messages could be leveraged if the choice is strategically informed by message content and the audience’s attributes (e.g., gender/marianismo and beneficence).

### Theoretical implications

Theoretically, this study expands the use of SDT, demonstrating the salience of autonomy, relatedness, competence, and beneficence. Competence is particularly relevant for connecting information needs within the COVID-19 and related vaccination context to being able to act. Additionally, given people’s desire to maintain autonomy, public health campaigns should carefully approach normative narratives and consider addressing specific information gaps within communities to equip the audience with the necessary knowledge to make informed decisions independently.

### Practical implications

We found softer approaches to vaccine storytelling with a human-interest focus that the audience feels connected to could be potentially more impactful in terms of attention and emotional engagement. Given many participants related to the character of the grandmother in the story and reflected on community-level inequalities, a narrative approach could be further supplemented by the prominent featuring of family and community interactions at the center of the COVID-19 vaccination decisions [48].

Practically, these findings can be applied in other vaccination contexts (e.g., HIV, routine childhood vaccinations). Given that routine childhood vaccinations declined for children born in 2020 and 2021 when compared with children born in 2018 and 2019 [49], it is important for public health officials, healthcare providers, and researchers alike to develop strategies and messaging to produce and share accurate vaccine-related information to improve immunization rates. Spanish-language media may be one method of reaching populations who need, and desire, this type of information yet do not have access.

### Limitations

Some limitations exist but provide a roadmap for future research. First, there is the potential loss of meaning due to some interviews being conducted in Spanish, transcribed, then translated into English prior to data analysis. While efforts were made to preserve participants’ original meaning (i.e., native Spanish-language speakers being involved in all stages from interviewing through data analysis) some nuances may be lost. Future research should continue these efforts. Second, the interviewers, while trained, sometimes missed opportunities for probing or follow-up questions, resulting in some of the original codes essentially being the questions and answers for some interviews. One interviewer also broke with protocol twice and presented the videos 1 and 2 back-to-back, then asked questions about both videos; similarly did the same for videos 3 and 4 instead of showing the videos and then asking the relevant questions. Continued training and interview checks will be necessary for future research. Some participants also brought a young child, which may have influenced their ability to fully pay attention.

Because our interviews addressed sensitive topics related to COVID-19, immigration, and health care, participants’ responses also may have been influenced by social desirability bias, or a tendency to report socially acceptable rather than fully candid views or behaviors. To minimize this risk, we emphasized confidentiality, conducted interviews in participants’ preferred language, used neutral, open-ended questions, and trained bilingual interviewers to adopt a nonjudgmental, rapport-building stance. Nonetheless, it is possible that some participants underreported stigmatized views or experiences, which should be considered when interpreting our findings.

## Conclusions

The goal of the present study was to investigate how ethnically minoritized individuals in a large county in Texas perceived video-based messaging aired on Spanish-language news media about COVID-19 vaccination, through the lens of self-determination theory. We found that immigration experiences and gender norms intersect to shape how people weigh family obligation against respect for others’ autonomy in vaccine decision-making. We also found that there is a significant need for accurate Spanish-language media. Spanish-language media that use human-centered, strategically framed messaging may offer a promising avenue for engaging this at-risk population and advancing health equity.

## Supporting information

S1 Text News report development. Description of the four news videos.
(DOCX)

S2 Text Interview guide. Interview questions.
(DOCX)
