# Supplementary material for: Perceptions of Spanish-language COVID-19 video messaging among the Hispanic community: A qualitative study in the United States of America
Source: PLoS One. 2026 Jul 29;21(7):e0339634. doi: 10.1371/journal.pone.0339634 (PMC13419170; doi:10.1371/journal.pone.0339634)
Supplement: S1 Text — Description of the four news videos. (DOCX) [file pone.0339634.s001.docx]

**S1 Appendix**

**News Report Development**

The interview study reported in this manuscript is a part of a larger project. Building on formative research conducted by the research team, we created a set of four news videos using specific frames based on the feedback from participants in the original focus groups. In designing these, the project team relied on framing, focusing on *loss* and *gain* frames, as well as the *individual* versus *collective* frames. The four news videos presented information about (1) multisystem inflammatory syndrome in children (MIS-C), a complication of COVID-19 infections first reported in 2020; (2) “long COVID” or the chronic continuation of symptoms after a COVID-19 infection; (3) an interview with a doctor about the COVID-19 vaccine, supplemented with statistics on hospitalization and mortality rates, and (4) a report on the protective effect of COVID-19 vaccines for seniors, with advice on how to secure a free vaccine. In the fourth video, for example, the news report was designed to highlight collectivistic frames (the impact on the broader community) using gain-frame messaging.
