# Supplementary material for: Perceptions of Spanish-language COVID-19 video messaging among the Hispanic community: A qualitative study in the United States of America
Source: PLoS One. 2026 Jul 29;21(7):e0339634. doi: 10.1371/journal.pone.0339634 (PMC13419170; doi:10.1371/journal.pone.0339634)
Supplement: S2 Text — Interview questions. (DOCX) [file pone.0339634.s002.docx]

# **Interview Questions**

**Purple = notes for interviewer**

**Blue = follow-up question as appropriate**

**BEGIN:**

- **Introduce yourself (UTA Student, etc.)**
- **Explain why you would be recording (so you can accurately note what was said)**
- **Explain that they will be kept anonymous, and no names will be used.**
- **Ask for their permission to record.**

**News Stories (COVID-19 in children; long Covid-19; reactions/need vaccine;**  **vaccines access)**

**Ask the first nine questions after each story is shown.**

1. What did you take away from this news story?

1. What did you think about the **/(1-4)reporters/(1-3)doctors/(2-3)anchors/ (last)public health** professional in the news stories? [**credibility/believability**]

1. What did you feel when watching the news stories?

1. Is there anything that would have improved your perception of **trust/credibility/believability**?

1. What did you think about the animations (**if present)**?

## How do you think your **family** might respond to the news story?

How do you think your **friends** might respond to the news story?

1. If you wanted to share the news stories, how would you do so (e.g., social media, direct text message, face to face)?
2. Did the news stories give you new information or change your thoughts about long-term COVID-19 effects/COVID-19 vaccine effects? ---- **How so?**
3. What would you like to see the news story talk more about?

**COVID-19 and Vaccines**

**Ask the following questions after all news stories have been shown and the above questions answered.**

1. What do you know about long COVID/long-term COVID-19 effects?

Do you have any concerns?

1. Where/from whom do you learn about the booster/vaccines?
2. What would you like to know more about?

1. What would encourage you or your family members to get the vaccine/booster if you haven’t already?

**What would discourage you?**

1. What would be important for you to know to make you more confident in the COVID-19 vaccine/boosters?

**What about for your children (if you have any)?**

1. What is your opinion of the COVID-19 vaccine/boosters?

**Has your opinion of the of the COVID-19 vaccine/boosters changed?**

**If yes, what prompted this change?**

**If no, why not?**

**15a. What do you see as the benefits of the ongoing COVID-19**

**boosters/vaccinations?**

**15b. What do you see as the obstacles of the COVID-19 boosters/vaccines? (next page)**

1. What other ways, beyond news stories, would you like to get health information? **(e.g., community health workers, doctors)?**

**From which sources do you like/prefer to get health information?**
